# Hydrothermal Fabrication of Silver Nanowires-Silver Nanoparticles-Graphene Nanosheets Composites in Enhancing Electrical Conductive Performance of Electrically Conductive Adhesives

**DOI:** 10.3390/nano6060119

**Published:** 2016-06-21

**Authors:** Hongru Ma, Jinfeng Zeng, Steven Harrington, Lei Ma, Mingze Ma, Xuhong Guo, Yanqing Ma

**Affiliations:** 1 School of Chemistry and Chemical Engineering, Shihezi University, Shihezi 832003, China; mahongru@stu.shzu.edu.cn (H.M.); zengjinfeng@stu.shzu.edu.cn (J.Z.); mamingze001@126.com (M.M.); guoxuhong@ecust.edu.cn (X.G.); 2 Tianjin International Center of Nanoparticle and Nanosystems, Tianjin University, Tianjin 300072, China; stevenharrington92@tju.edu.cn (S.H.); maleixinjiang@tju.edu.cn (L.M.); 3 State Key Laboratory of Chemical Engineering, East China University of Science and Technology, Shanghai 200237, China; 4 Key Laboratory for Green Processing of Chemical Engineering of Xinjiang Bingtuan, Shihezi University, Shihezi 832003, China; 5 Engineering Research Center of Materials–Oriented Chemical Engineering of Xinjiang Bingtuan, Shihezi University, Shihezi 832003, China

**Keywords:** hydrothermal, nanosilver, graphene, resistivity, electrically conductive adhesives

## Abstract

Silver nanowires-silver nanoparticles-graphene nanosheets (AgNWs-AgNPs-GN) hybrid nanomaterials were fabricated through a hydrothermal method by using glucose as a green reducing agent. The charge carriers of AgNWs-AgNPs-GN passed through defect regions in the GNs rapidly with the aid of the AgNW and AgNP building blocks, leading to high electrical conductivity of electrically conductive adhesives (ECA) filled with AgNWs-AgNPs-GN. The morphologies of synthesized AgNWs-AgNPs-GN hybrid nanomaterials were characterized by field emission scanning electron microscope (FESEM), and high resolution transmission electron microscopy (HRTEM). X-ray diffraction (XRD) and laser confocal micro-Raman spectroscopy were used to investigate the structure of AgNWs-AgNPs-GN. The resistance of cured ECAs was investigated by the four-probe method. The results indicated AgNWs-AgNPs-GN hybrid nanomaterials exhibited excellent electrical properties for decreasing the resistivity of electrically conductive adhesives (ECA). The resistivity of ECA was 3.01 × 10^−4^ Ω·cm when the content of the AgNWs-AgNPs-GN hybrid nanomaterial was 0.8 wt %.

## 1. Introduction

Metal nanomaterials have attracted a great deal of research attention and have tremendous potential in a variety of high-performance antibacterial applications [1], catalysts [2,3,4,5], sensors [6,7,8], optoelectronic devices and electronics [9,10,11]. Metal-based nanomaterials usually have excellent thermal, mechanical and electrical properties [12] compared to conventional materials. Particularly, one-dimensional (1D) nanomaterials such as nanotubes [13,14], nanowires [15,16] and nanorods [17,18] fulfill an important role in the development of conductive nanomaterials. The higher aspect ratio and smaller dimensions of 1D nanomaterials allow them to effectively transport electrical carriers along one controllable path and they have demonstrated that an increase in their aspect ratios will ultimately increase the electrical conductivity of composites [19,20]. It is expected that distinct properties from the 1D nanomaterials will be exhibited in the metal-based nanomaterials’ hybrid nanomaterial. Many kinds of 1D nanomaterials have been investigated to produce conductive composites, especially AgNWs, and they have drawn prodigious interest for several reasons, such as their stable chemical properties and high electrical conductivity [21,22]. Furthermore, random networks of AgNWs are promising candidates for conductive devices. As a result, AgNWs have been examined for potential use in many applications including chemical and biological sensors and electrically conductive adhesives (ECAs).

For this paper, silver nanoparticles (AgNPs) were introduced into ECAs. This is because AgNPs can fill gaps in two possible ways. In the first, some of the conductive paths are formed by filling gaps between linked AgNWs. In the second, more conductive paths can be formed by filling the gaps between unattached silver flakes. In addition, AgNWs may improve the conductivity of ECAs in yet another way. They are excellent materials to supply junctions for wire-built networks, and so the number of conductive pathways can be increased and the effective contact area can be enlarged. The contact resistance of AgNWs is less than that of AgNPs in ECA samples. Therefore, the conductivity of ECAs is improved in the presence of both AgNWs and AgNPs. Taking this information into account, the conductivity of ECAs with a mixture of AgNWs-AgNPs-GN is better than that of ECAs containing only AgNPs-GN.

Regardless of whether AgNWs or AgNPs are used, both tend to coalesce during applications due to their high surface energies and van der Waals forces [23]. However, silver-graphene hybrid nanomaterials are very fascinating materials that combine the key features of silver and graphene. In this paper, AgNWs-AgNPs-graphene nanosheets hybrid nanomaterial is used as conductive filler in ECA instead of partial silver flakes. The electrical conductivity of the ECA can be increased by the inclusion of conductive fillers into the insulating resin matrix. Graphene has drawn interest as well because of its large surface area and high electrical conductivity [24,25,26,27]. However, graphene synthesis from graphene oxide introduces a considerable amount of defects, reducing electrical conductivity [28,29]. This work demonstrates that a system containing AgNWs and AgNPs is an efficient way to provide the electronic transmission channels of Ag-Ag junctions on the surface of graphene [30], while simultaneously decreasing the resistivity of the ECA containing AgNWs-AgNPs-GN hybrid nanomaterials.

## 2. Experimental Section

### 2.1. Materials

Natural graphite flakes (325 mesh) were obtained from Alfa Aesar. Hydrochloric (HCl, 38%) and hydrogen peroxide (H_2_O_2_, 30%) were purchased from Tianjin Fuyu Fine Chemical Co. Ltd. (Tianjin, China). Potassium permanganate (KMnO_4_, ≥99.5%) and glucose were supplied by Tianjin Shengyu Chemical Co. Ltd. Sodium nitrate (NaNO_3_, ≥99.0%) was supplied by Aladdin industrial Co. (Shanghai, China). Concentrated sulfuric acid H_2_SO_4_ (>98%) was obtained from Chengdu Area Kelong Chemical Co. Ltd. (Chengdu, China). Silver nitrate (AgNO_3_, >99.8%) was purchased from Xi’an Chemical Co. (Xi’an, China). Polyvinylpyrrolidone (PVP, K30) and sodium chloride (NaCl, AR) were obtained from Tianjin Guangfu Fine Chemical Research Institute. Glycerolwas obtained from Tianjin Zhiyuan Chemical Reagent Co. Ltd. (Tianjin, China). Epoxy resin (862), used as epoxy binder was purchased from Guangzhou Topyon Trade Co. Ltd. (Guangzhou, China). The catalysts 1-(2-Cyanoethyl)-2-ethyl-4-methylimidazde (2E4MZ-CN) and 4-Methylcyclohexane-1, 2-dicarboxylic Anhydride (MHHPA) were obtained from TCI (Shanghai) Development Co. Ltd. The micro silver flakes were purchased from Strem Chemicals, Inc. (Newburyport, MA, USA). All reagents and solvents were used without further purification.

### 2.2. Synthesis of AgNWs

The AgNWs were synthesized in ways similar to what has already been described in literature [31]. Briefly, 5.86 g PVP was slowly added into 190 mL glycerol in a three-necked round bottle flask until all PVP was dissolved with gentle mechanical stirring (50 rpm) and heating. After cooling, AgNO_3_ (1.58 g) was added into the mixed solution. The flask was then immersed into oil bath. The glycerol solution (10 mL) of NaCl (59 mg) and H_2_O (0.5 mL) was added into the three-necked round bottle flask. The mixture was heated to 160 °C. When the temperature reached 160 °C, heating ceased and the mixture was allowed to cool to room temperature. Gentle mechanical stirring (50 rpm) was constantly applied during the entire process of AgNWs growth. After, the obtained product was washed with water through centrifugation, and subsequently re-dispersed in water.

### 2.3. Preparation of Graphene Oxide and AgNWs-AgNPs-GN

Graphene oxide (GO) was prepared using a modified Hummers method [32]. In a typical procedure, the glucose (0.08 g) was added to graphene oxide (60 mL, 1 mg/mL) under stirring. Then 90 mg AgNWs were added to above solution with ultra-sonication for 1 h, and the mixture solution was heated in the stainless steel autoclave with a Teflon liner of 100 mL capacity under the condition of 180 °C for 18 h. After the autoclave was air-cooled to room temperature unaided, the product was isolated by centrifugation and washed with distilled water, then dried by lyophilization for characterization and experiments.

### 2.4. Preparation of the ECA Based on AgNWs-AgNPs-GN

The polymer matrix contains epoxy resin, 2E4MZ-CN and HMMPA in a weight ratio of 1:0.0185:0.85. The content of AgNWs-AgNPs-GN hybrid nanomaterial varies from 0.0%, 0.2%, 0.5%, 0.8% to 1.1%, and 30% polymer matrix. Two strips of polyimide adhesive tape were affixed to the surface of a cleaned glass slide separated by a distance of 3 mm. The uncured ECA mixture was bladed uniformly into the gap between the two strips. The polyimide tapes were removed from the glass slide when the ECA was cured at a temperature of 150 °C for 2 h.

## 3. Characterization of AgNWs-AgNPs-GN

The dispersion and morphology of the AgNWs-AgNPs-GN were characterized by field emission scanning electron microscopy (FE-SEM) on a Hitachi Limited (Tokyo, Japan) and high resolution transmission electron microscopy (HR-TEM) on a FEI Tecnai G20 (FEI, Hillsboro, OR, USA). The X-ray diffraction spectra (XRD) measurements were performed using a Bruker D8 advanced X-ray diffractometer (Bruker, Germany) with Cu Kα irradiation (λ = 1.5406 Å) in the scanning angle range of 10°–90° at a scanning rate of 10°/min at 40 mA and 40 kV, which was used to investigate the crystalline structure of the AgNWs-AgNPs-GN. The Raman spectra of AgNWs-AgNPs-GN were obtained using laser confocal micro-Raman spectroscopy (LabRAM HR800, Horiba Jobin Yvon, Paris, France) equipped with a 532 nm laser. X-ray photoelectron spectroscopy (XPS) was completed with a monochromatic MgKαX-ray source (*hv* = 1486.6 eV) on an AMICUS/ESCA 3400 (Shimadzu, Japan), which was used to analyze the elements and the functional groups on the surface of AgNWs-AgNPs-GN. The thickness of the cured ECA was measured by a thickness gauge (Shanghai Chuanlu Measuring Tool Limited Liability Co., Shanghai, China). Resistance was measured by the RTS-8 four point probe Resistivity Measurement system (Guangzhou, China).

## 4. Results and Discussion

**Figure 1:**
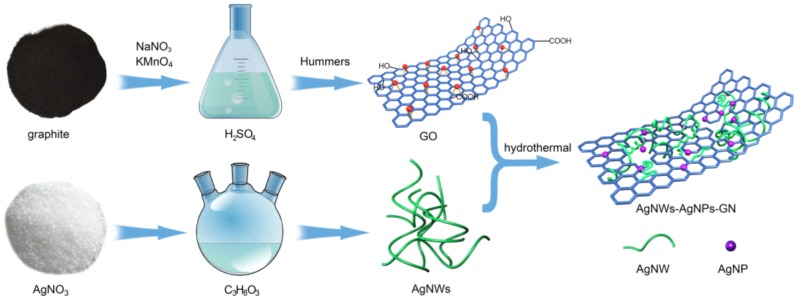
Schematic of preparing the silver nanowires-silver nanoparticles-graphene nanosheets (AgNWs-AgNPs-GN).

The synthesis of the AgNWs-AgNPs-GN hybrid nanomaterial is illustrated in Figure 1. GO was synthesized according to a modified Hummers method and dispersed in water by ultrasonic treatment to form a brown suspension. The AgNWs were prepared via reduction by glycerol. Then an appropriate amount of AgNWs was added into the GO aqueous dispersion and sonicated for 2 h to achieve a homogeneous mixture. Next, the mixture containing a certain amount of glucose was added to a stainless steel autoclave with a capacity of 100 mL and Teflon liner. The color of the mixture changed from brown to black, indicating that GN was obtained. The AgNWs were fused and produced some AgNPs due to the high reaction temperature, but as a result the lengths of the AgNWs were reduced. Fortunately, the AgNPs filled the gaps among the AgNWs and formed the conductive network.

**Figure 2:**
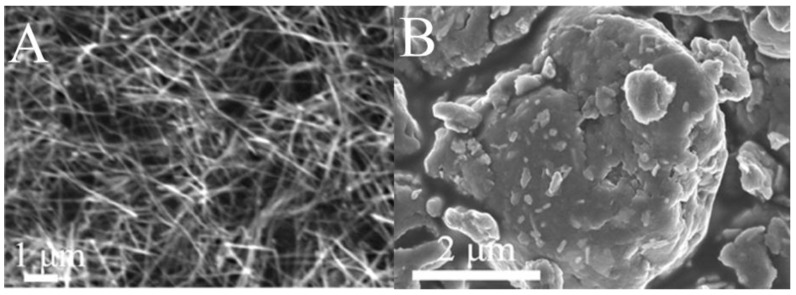
Field emission scanning electron microscopy (FE-SEM) images of the AgNWs (**A**) and AgNWs-AgNPs-GN (**B**).

The AgNWs as shown in Figure 2A possessed an average length of about 4 μm and a diameter of about 50 nm. A large number of AgNWs agglomerated due to high surface energy. There was an irregularly large number of AgNWs and AgNPs that coexisted on the GN layers observed from Figure 2B, and the agglomeration of AgNWs decreased distinctly.

**Figure 3:**
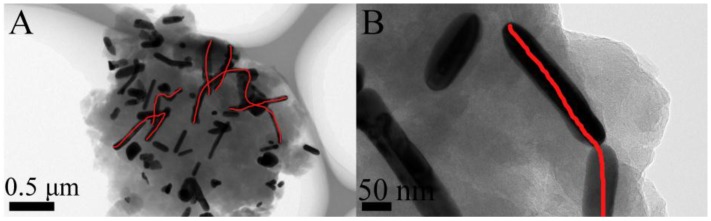
High resolution transmission electron microscopy (HRTEM) images of the AgNWs-AgNPs-GN (**A**) and magnification of one segment of the AgNWs-AgNPs-GN (**B**).

Figure 3 present the high resolution transmission electron microscopy (HRTEM) images of the synthesized AgNWs-AgNPs-GN hybrid nanomaterial with different magnifications. It can be seen that some AgNWs and AgNPs were present on the surface of the GN. The average diameter of the AgNWs was 55 nm and their lengths ranged from 100 nm to 500 nm. The average diameter of the AgNPs was measured to be 90 nm. In addition, it can be observed that AgNWs and AgNPs are in the same plane and form conductivity networks on the surface of the GN (denoted by the red lines). These conductivity networks may improve the electrical properties of AgNWs-AgNPs-GN hybrid nanomaterial and further enhance the electrical properties of ECAs containing AgNWs-AgNPs-GN hybrid nanomaterial.

**Figure 4:**
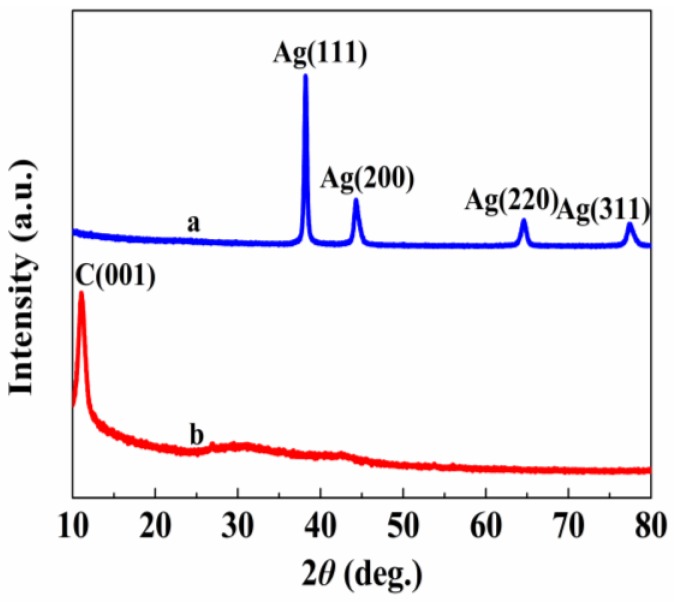
X-ray powder diffractometer (XRD) patterns of graphene oxide (GO) and AgNWs-AgNPs-GN (a: AgNWs-GNs, b: GO).

The crystal structures of the AgNWs-AgNPs-GN hybrid nanomaterial were characterized using an X-ray powder diffractometer (XRD) (Figure 4). It can be seen that there is an intense and sharp diffraction peak in Figure 4b at 2θ = 11.09°, which is attributed to the (001) lattice plane of GO. An interlayer d-spacing between the GO nanosheets can be calculated at 0.80 nm. This indicated the oxygen-containing functional groups were introduced into the GO layers [33]. For AgNWs-AgNPs-GN hybrid nanomaterials (Figure 4a), the characteristic diffraction peak of GO disappeared, indicating that the GO was reduced through a hydrothermal method by using glucose as a reducing agent. The peaks at 2θ = 38.17°, 44.24° and 64.57° can be assigned to the (111), (200) and (220) crystalline planes of silver, respectively [34]. These peaks match well with the data from the PDF card (PDF2-2004) for silver. The XRD results show that the silver was present on the surface of the GN, which corresponded to what was observed via SEM and TEM.

**Figure 5:**
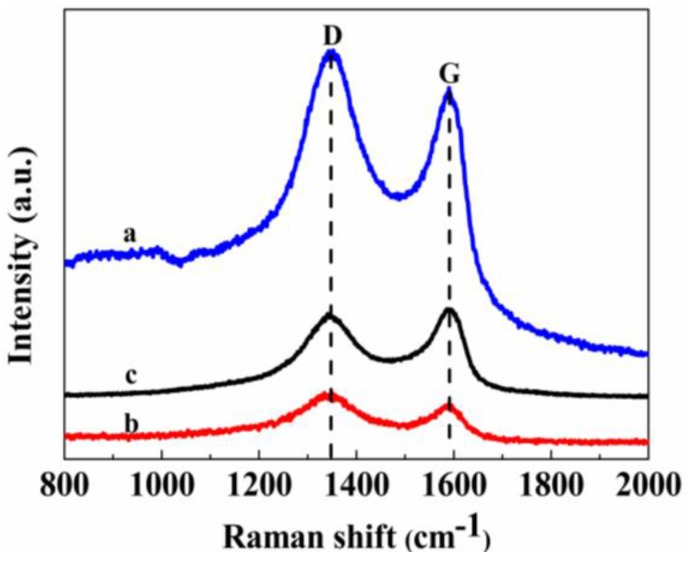
Raman spectra of GO, GN and AgNWs-AgNPs-GN (a: AgNWs-GNs, b: GO, c: GN).

Raman spectroscopy is very sensitive to the microstructure of nanomaterials, and so it was used to illustrate the GN crystalline structure. As shown in Figure 5, the D band at 1351.3 cm^−1^ represents structural defects related to the size of the sp^2^ domains [5,35]. The G band at 1589.6 cm^−1^ corresponds to the ordered sp^2^ hybridized graphene structure [36]. The relative intensity ratio of the D and G band elucidates the disorder degree of GN. It was calculated that the *I*_D_/*I*_G_ ratio was 1.11 for AgNWs-AgNPs-GN (Figure 5a), 1.14 for GO (Figure 5b) and 0.86 for GNs (Figure 5c). The increase of the *I*_D_/*I*_G_ ratio of AgNWs-AgNPs-GN is due to the incorporation of AgNWs-AgNPs, in which the AgNWs-AgNPs graft on the surface of the GNs and replace some of the sp^2^ carbon sites and generate more sp^3^ carbon forms. The intensity ratio of *I*_D_/*I*_G_ of both the AgNWs-AgNPs-GN and the GN decreases compared to that of GO, suggesting there is a decrease of oxygen-functional groups on GO.

**Figure 6:**
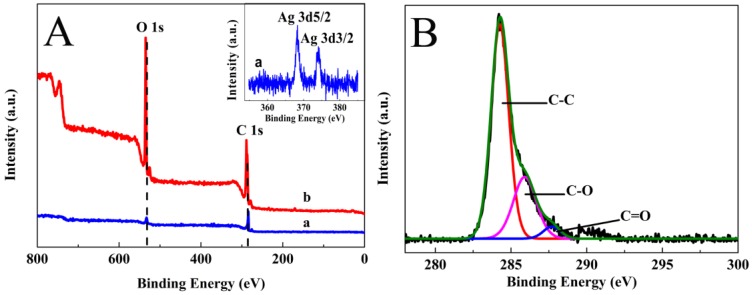
X-ray photoelectron spectroscopy (XPS) wide-scans of AgNWs-AgNPs-GN (a) and GO (b), insert: magnification of segment of a line (**A**) and C 1s narrow-scans XPS spectra of AgNWs-AgNPs-GN (**B**).

XPS was used to provide further insight into the nanostructure of AgNWs-AgNPs-GN and GO, the results of which are shown in Figure 6. The peaks of the elements C and O appeared at 287.6 and 533.1 eV, respectively. The C, O atomic mass ratio of AgNWs-AgNPs-GN was 5.46, which was a significant increase compared to that of GO (1.78). This ratio indicated that the oxygen-containing functional groups on the surface of the GO were effectively removed via a hydrothermal method. The insert is the XPS signature of the Ag 3d doublet (3d_5/2_ and 3d_3/2_) for the AgNWs-AgNPs deposited on the rGO. The Ag 3d_5/2_ and 3d_3/2_ peaks of AgNWs-AgNPs-GN nanocomposites appeared at 368.3 and 374.3 eV, respectively. The spin-orbit splitting of doublet components for Ag 3d_5/2_ and 3d_3/2_ was calculated to be 6 eV, indicating the presence of Ag in the hybrid nanomaterial [37].

The C 1s XPS spectrum of GN exhibits oxidation with three components of energy to 284.3, 285.9 and 287.7 eV, corresponding to the C–C, C–O, and –C=O groups, respectively. The functional group mass was 69.92% for C–C, 26.28% for C–O, 3.80% for –C=O. The mass of C–C was much higher than that of GO [38], providing further evidence that oxygen-containing functional groups were effectively removed.

## 5. Measurements of Electrical Properties

The uncured ECAs were bladed into five identical specimens. Five points for each specimen were selected after the ECA was cured at 150 °C for 2 h. The resistance of these five points was measured by the four-point probe resistivity measurement system. Then the resistivity of ECAs was calculated using Equation (1).

(1) $$\rho =R_{L}\times T$$

where ρ is the calculated resistivity value of cured ECA (Ω·cm); *R_L_* issquare resistance (Ω); and *T* is the thickness of cured ECA (cm).

**Figure 7:**
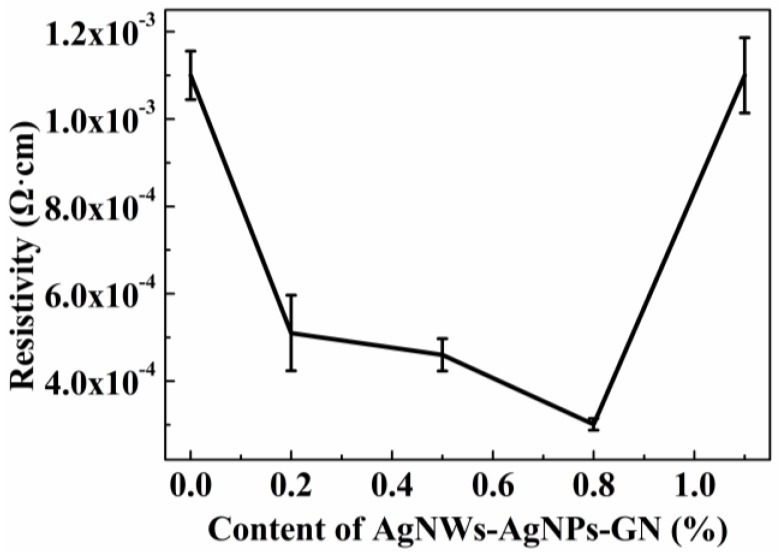
Resistivity of electrically conductive adhesives (ECA)-changing trend image under different contents of AgNWs-AgNPs-GN.

**Figure 8:**
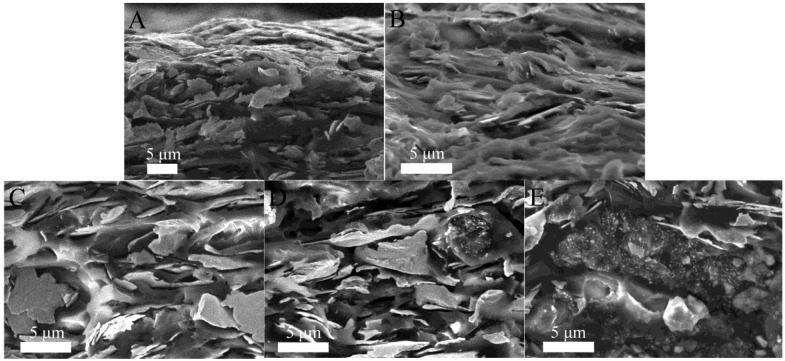
SEM images of cross-section morphology of electrically conductive adhesives (ECA) filled with 0.0 wt % AgNWs-AgNPs-GN (**A**), 0.2 wt % AgNWs-AgNPs-GN (**B**), 0.5 wt % AgNWs-AgNPs-GN (**C**), 0.8 wt % AgNWs-AgNPs-GN (**D**), and 1.1 wt % AgNWs-AgNPs-GN (**E**).

The ECAs were prepared via mixing matrix resin and silver flakes. AgNWs-AgNPs-GN was introduced into the mixture in order to fill the gaps between the unattached silver flakes, as well as to build a new conductivity network in the matrix resin. The resistivity of ECAs containing AgNWs-AgNPs-GN decreased with the increase of the AgNWs-AgNPs-GN content, which was observed from Figure 7. The resistivity of the ECAs dipped as low as 3.01 × 10^−4^ Ω·cm when the content of AgNWs-AgNPs-GN reached 0.8%. However, the resistivity of the ECAs significantly increased after the content of AgNWs-AgNPs-GN surpassed 0.8%. One cause of this phenomenon is that AgNPs filled the gaps between the AgNWs, and the AgNWs-AgNPs formed a random conductive network on the surface of graphene which improved the number of conductive paths. Therefore, the resistivity of the AgNWs-AgNPs-GN hybrid nanomaterial was decreased (as shown in Figure 8). Another cause is that the conductive networks are formed in matrix resin due to AgNWs-AgNPs-GN linking to unattached silver flakes, or they connect each other. However, excess AgNWs-AgNPs-GN led to a significant increase of resistivity of ECAs and resulted in electrical performance deterioration. When the content of AgNWs-AgNPs-GN filler increased to 1.1 wt %, the resistivity of the ECAs increases and the conductivity performance goes down [39]. The optimal content of AgNWs-AgNPs-GN is 0.8 wt %. The attractive interactions between conductive fillers contributed to generating an agglomeration-free conductive network and a more conductive channel [40].

As shown in the SEM images of the cross-section of cured ECAs in Figure 8, for the ECA filled with silver flakes only, there were many gaps among the silver flakes providing more electrical pathways. The AgNWs-AgNPs-GN can be inserted between the gaps of silver flakes when a small amount of AgNWs-AgNPs-GN is added. It can also be observed that the conductive fillers were enwrapped by the epoxy resin without agglomerating and adhering into the blocks seen in Figure 8B,C. The aggregation began to form when the content of AgNWs-AgNPs-GN reached 0.8%. This provided a desirable environment for the uniform distribution of the conductive fillers, and a favorable conductive network was formed. However, when a great quantity of AgNWs-AgNPs-GN was added, the AgNWs-AgNPs-GN at a high content easily agglomerated as shown in Figure 8D,E, which created a large number of contact points between the silver flakes, increasing the tunneling resistance and further decreasing the electric properties of the ECAs.

## 6. Conclusions

Electrically conductive adhesive incorporated with AgNWs-AgNPs-GN can be prepared with high electrical conductivity. In this study, AgNWs-AgNPs-GN nanomaterial was chosen as a nanofiller and exhibited a well-dispersed morphology and decreased the electrical resistivity of the electrically conductive adhesive. A physical network formation of AgNWs-AgNPs was indicative of when the AgNWs-AgNPs-GN content was increased. The electrically conductive adhesive prepared by 69.2% silver flakes and 0.8% AgNWs-AgNPs-GN reached an electrical resistivity of 3.01 × 10^−4^ Ω·cm. It is expected that this electrically conductive adhesive with AgNWs-AgNPs-GN will be a promising material for the development of electronic packaging in the near future.
